# Buried Defect Detection Method for a Blowout Preventer Seal Ring Groove Based on an Ultrasonic Phased Array

**DOI:** 10.3390/ma15186429

**Published:** 2022-09-16

**Authors:** Shiqiang Wang, Laibin Zhang, Peihang Yu, Qiang Xu, Jianchun Fan, Jiamin Yu

**Affiliations:** 1 College of Safety and Ocean Engineering, China University of Petroleum (Beijing), Beijing 100100, China; 2 Research Institute of Safety, Environmental Protection and Quality Supervision and Inspection, Chuanqing Drilling Engineering Co., Ltd., Guanghan 618300, China

**Keywords:** blowout preventer, gasket ring groove, ultrasonic phased array detection, structural echo, defect echo

## Abstract

This study aims to investigate an accurate detection method to detect defects in the gasket ring groove of the blowout preventer (BOP) using the ultrasonic phased array technology. Traditionally, it is difficult to accurately determine the type and size of defects in the gasket ring groove due to the complexity of the BOP configuration and the interference between the defect echo and the structural echo when using the ultrasonic phased array detection technology. In this study, firstly, the appropriate detection process parameters are determined by using simulation software for simulating and analyzing the defects of different sizes and types in the gasket ring groove of a BOP. Thereafter, according to the detection process parameters determined by the simulation analysis, we carry out a corresponding actual detection test. Simulation analysis and detection test results show that the relative amplitude of the test results and the simulation results differ within 1 dB, and the simulation results have a guiding role for the actual detection. The defect echo and structure echo can be clearly distinguished by selecting appropriate detection process parameters, such as probe frequency 5 MHz, array elements 36, and probe aperture 16 mm. The research results can provide theoretical reference for the detection of blowout preventer.

## 1. Introduction

**Figure 1:**
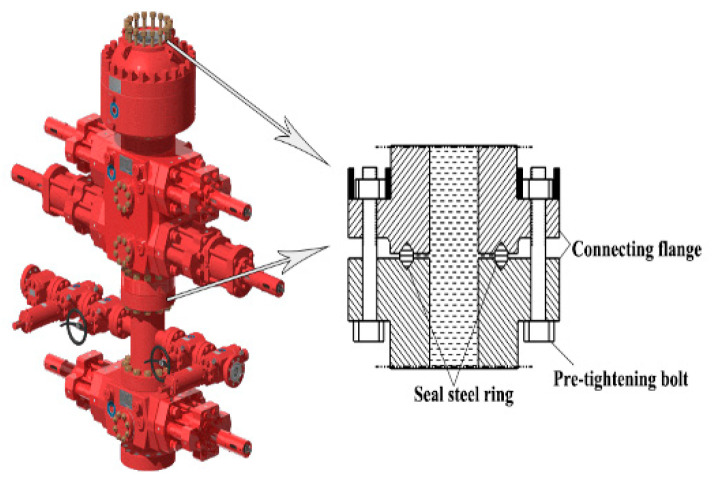
Schematic diagram of the sealing connection structure of a BOP stack.

The blowout preventer stack, including an annular BOP, a single-ram BOP, and a double-ram BOP, is a safe wellhead sealing device used to prevent blowout during drilling, workover, and oil testing [1]. The BOP stack is bolted together, and metal seals are installed in the ring groove to prevent leakage. A schematic diagram of the sealing connection structure of the BOP system is shown in Figure 1.

**Figure 2:**
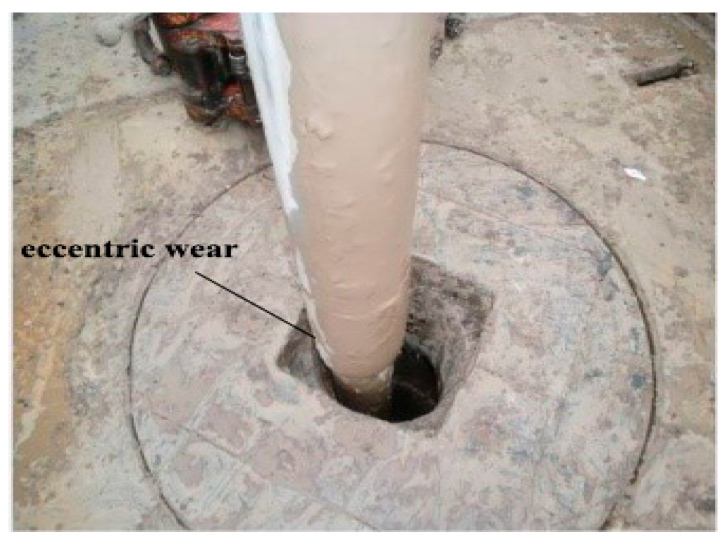
Off-center drill string on site.

The BOP stack is installed on the wellhead casing, and the derrick is installed in the center of the wellhead to avoid wellhead misalignment during oil drilling. However, the drill string rotates and deviates from the center of the wellhead during drilling, as shown in Figure 2.

**Figure 3:**
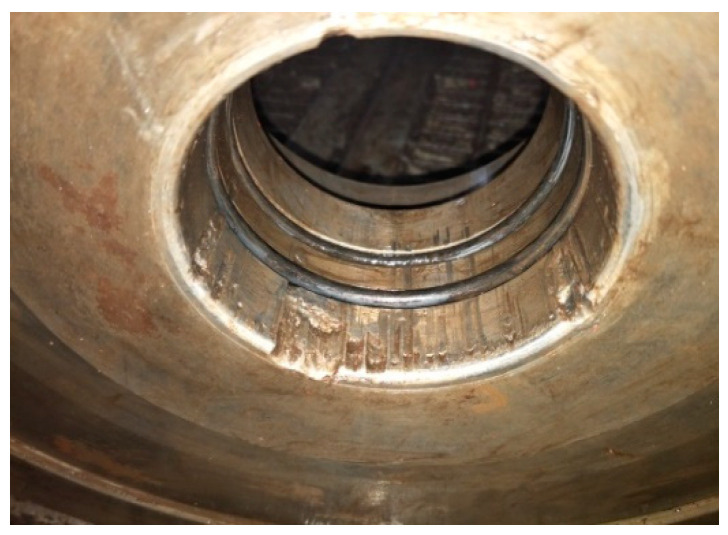
Eccentric wear phenomenon on the inner wall of the main diameter of a BOP.

During workover operations, the long drill string is rotated while drilling, resulting in collisions between the drill string and the BOP. Wear can occur between the inside of the BOP and the outside of the drill pipe during these collisions. This can cause eccentric wear on the BOP main bore wall. In more severe cases, this can cause damage to the metal seal ring groove of the BOP main bore, as shown in Figure 3, resulting in difficulty in successfully sealing the BOP stack. When the BOP cannot be effectively sealed, a blowout accident can occur, generating great economic loss and environmental pollution.

**Figure 4:**
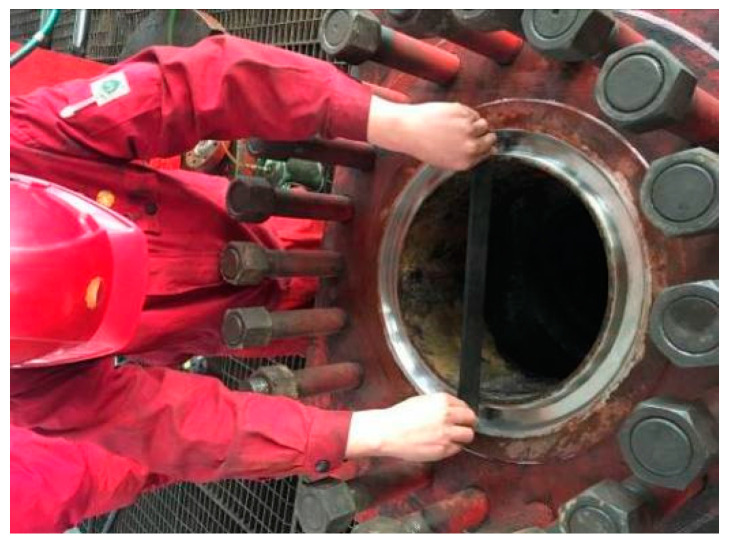
Eccentric wear measurement of the metal sealing ring.

Therefore, in addition to considering the mechanical design and manufacturing of the BOP [2,3], based on the above failure analysis of the metal seal part of a BOP, the eccentric wear amount of the metal seal ring groove can be accurately measured during regular inspection and maintenance. Regions of the seal ring groove damaged by eccentric wear can be repaired by surface welding, as shown in Figure 4. However, defects such as porosity, slag inclusion, cracks, and incomplete welding may exist after the surface welding process. Therefore, it is necessary to conduct nondestructive testing on the welding part of the main bore seal of the BOP to prevent seal failure. Magnetic particle and penetration methods can be used to detect surface cracks on the welding part of the BOP main bore metal seal; however, there is no good method to detect internal buried defects. Tang et al. [4] conducted simulations and experiments on the stress distribution of a 2FZ54-105 double-ram-type BOP. Their results showed that stress concentrations exist on the internal surface of the valve body near the intersection of the stamping cavity and the vertical hole, the flange neck at the intersection of the flange and the valve body, the connection between the small flange neck and the valve body, and the edge of the arc surface in the stamping cavity. These stress concentration regions are the weak points of the BOP. Due to the high strength requirements for the gasket ring groove, surface welding is often used for manufacturing the gasket ring groove, but this welding technique can produce defects such as porosity, slag inclusion, and cracks [5]. Welding defects can greatly affect the mechanical properties of the material [6], causing a serious impact on the safety seal of the BOP. Detecting internal defects in a BOP gasket ring groove quickly and rigorously is a problem that urgently needs to be solved.

At present, the commonly used nondestructive testing methods for welding defects include acoustic emission testing [7], magnetic particle testing [8], penetrant testing [9], eddy current testing [10], radiographic testing [11], and pulsed thermography testing [12]. Acoustic emission detection is based on the acoustic emission phenomenon of solid materials. When a solid material undergoes local elastoplastic deformation, cracking, phase change, and other dynamic changes under the actions of external and internal forces, it quickly releases energy in the form of stress waves [13]. Magnetic particle testing detects magnetic flux leakage caused by defects in the material using small magnetic particles with fluorescent properties. Magnetic particle testing is commonly used to detect surface and subsurface defects in ferromagnetic materials such as castings, forgings, rolled steel plates, heat-treated parts, and machined and ground parts [14]. Liquid penetrant inspection employs the inherent accumulation of a fluid around a discontinuity to create noticeable indication at the surface defects of parts manufactured from nonporous materials. The capillary effect arising from surface tension due to cohesive force between the molecules of the liquid and wetting properties of the material causes the liquid to penetrate the openings on the surface [15]. The principle of eddy current detection is based on electromagnetic induction. Eddy current detection has the benefit of lower cost and higher detection efficiency and is very sensitive when detecting surface and near-surface defects [16]. The radiographic method is based on the partial absorption of penetrating radiation passing through the object under investigation. The radiographic method has been widely adopted to nondestructively examine potential defects of welded joints in pipes [17]. Defects such as cracks within the theoretical skin depth range can disturb the eddy current density distribution, resulting in a higher level of Joule heating in the area where the current density increases, thereby affecting the local temperature distribution. Therefore, defects can be detected from thermal images [18], and this method is called pulsed thermography.

However, the detection methods mentioned above can only be used to detect surface defects and cannot be well applied to detect buried defects in the seal ring groove of a BOP. The ultrasonic phased array detection method has been widely used to detect internal defects of different workpieces due to its advantages of strong penetrability, high positioning accuracy, and rapid detection [19,20,21]. Feng et al. [22] coupled the ultrasonic wave reflection principle and image processing to enhance sizing surface cracks in welded tubular joints using an ultrasonic phased array. This approach reduced the detection errors of the crack size and orientation due to different legs and varied probe locations. Lopez et al. [23] used an ultrasonic phased array to detect the defects of aluminum parts in additive manufacturing and confirmed that ultrasonic phased array detection technology can be applied to detect defects in additive manufacturing parts. Ma et al. [24] presented a method based on signal correlation to detect delamination defects in widely used carbon-fiber-reinforced plastic to distinguish defect and nondefect signals. This method required less inspector prior knowledge compared to the ultrasonic C-scan method. Experimental results showed that the defect size error was less than 4% and the depth error was less than 3%. Li et al. [25] studied the ultrasonic phased array detection method by detecting defects in butt welds on the complex surface parts of engine blades. Their research results showed that the finite element method is an effective tool to study the ultrasonic phased array detection of complex surfaces. Wang et al. [26] presented an effective nondestructive method using phased array ultrasonic testing to characterize submillimeter artificial deep bottom holes in additive manufactured TC18 titanium blocks. Their results showed that the annular array has a higher detection accuracy than the linear array PAUT. Chabot et al. [27] proposed a comprehensive control method to conduct in situ ultrasonic control for direct energy deposition. They particularly illustrated the defect detection of wire-arc additive manufacturing components and laser metal deposition using the phased array ultrasonic testing technique. The above research works showed that it is more efficient to detect defects by combining finite element analysis and experimental detection when using ultrasonic phased array technology.

Nondestructive testing methods, such as acoustic emission testing, magnetic particle testing, and eddy current testing, cannot well detect the size and location of defects in the gasket ring groove of a BOP. Therefore, we specialize in ultrasonic testing and evaluation of structural damage of petroleum equipment. This paper initially used the finite element method to simulate the ultrasonic phased array detection process of a BOP gasket ring groove and then carried out the corresponding test according to the finite element simulation analysis results. The results showed that ultrasonic phased array detection technology can effectively detect internal defects in the gasket ring groove of a BOP.

## 2. Identification Method of Structure Echo and Signal Echo Using Ultrasonic Phased Array

### 2.1. Finite Difference Model of Acoustic Waves in an Elastic Solid

It is particularly important to explore the propagation mode of ultrasonic waves in elastic solid media and improve the detection accuracy of abnormal structures. This is because of the complexity of waveform conversion of ultrasonic wave propagation in complex abnormal structures. The finite difference method can be used to simulate various complex structures and characterize the difference between the structure echo and the signal echo. Then, a time-domain broadband simulation of complex signals can be realized through step-by-step method calculation, which can more vividly describe the change in ultrasonic waves in space or the change in waveform at a certain point. In this paper, two-dimensional acoustic wave equations are used to describe the sound field characteristics of elastic solids by the finite difference time-domain method. The acoustic wave equation is [28]:

(1) $$\mu \nabla^{2}\vec{U}+\left(\lambda +\mu \right)\nabla \theta =\rho \frac{\partial^{2}\vec{U}}{\partial t^{2}}$$

(2) $$\nabla^{2}=\frac{\partial^{2}}{\partial x^{2}}+\frac{\partial^{2}}{\partial y^{2}}+\frac{\partial^{2}}{\partial z^{2}}$$

(3) $$\theta =\frac{\partial u}{\partial x}+\frac{\partial v}{\partial y}+\frac{\partial w}{\partial z}$$

In the formula, ∇^2^ is the Laplace operator; *θ* is the divergence of the displacement; $\vec{U}$ is the displacement of the mass point; *ρ* is the density of the medium; and the properties of the elastic solid are expressed by the elastic constants *λ* and *μ*.

In the two-dimensional rectangular coordinate system, the component form of the acoustic wave Equation (1) is:

(4) $$\{\begin{array}{c} \frac{\partial^{2}u_{x}}{\partial t^{2}}=c_{L}^{2}\frac{\partial^{2}u_{x}}{\partial x^{2}}+\left(c_{L}^{2}-c_{S}^{2}\right)\frac{\partial^{2}u_{z}}{\partial x\partial z}+c_{S}^{2}\frac{\partial^{2}u_{x}}{\partial z^{2}}\\ \frac{\partial^{2}u_{z}}{\partial t^{2}}=c_{S}^{2}\frac{\partial^{2}u_{z}}{\partial x^{2}}+\left(c_{L}^{2}-c_{S}^{2}\right)\frac{\partial^{2}u_{x}}{\partial x\partial z}+c_{L}^{2}\frac{\partial^{2}u_{z}}{\partial z^{2}} \end{array}$$

In the formula, *C_L_* is the longitudinal wave velocity, and *C_S_* is the shear wave velocity.

### 2.2. Deflection and Focusing of Phased Array

**Figure 5:**
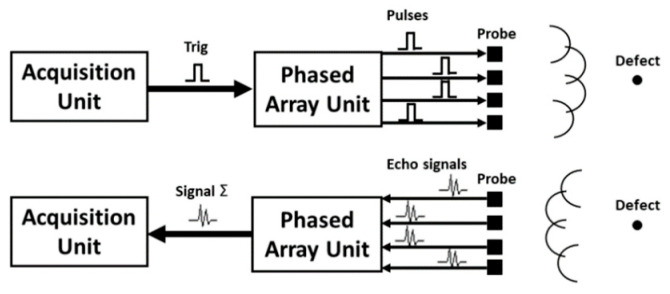
Delay rule of the ultrasonic phased array.

The transducer in the ultrasonic phased array includes multiple piezoelectric elements that can be controlled and receive feedback signals individually. The relative delay of the excitation signal between different crystals in the probe can be changed by deflecting the sound beam in different directions without changing the motion state of the phased array. At the same time, phased array technology can focus the sound beam by applying a nonlinear delay law to it, as shown in Figure 5. Therefore, the beam can be deflected and focused at the same time using a complex combination of these delay rules. It is necessary to apply different chip delay times at finite difference discrete points. If the chip excitation signal is a three-period sine wave plus a Hanning window signal:

(5) $$f\left(t\right)=\{\begin{array}{c} {}[1-\cos(2\pi f(t-t_{j})/3]\cos(2\pi f(t-t_{j})),\;\;\;\;\;\;\;\;\;\;\;0\leq t\leq \frac{f}{3}\\ 0,\;\;\;\;\;\;\;\;\;\;\;\;\;\;\;\;\;\;\;\mathrm{others} \end{array}$$

The signal after applying the delay term *t_j_* is [29]:

(6) $$t_{j}=\frac{F}{c}\left\{{\left[1+{\left(\frac{Nd}{F}\right)}^{2}-2\frac{Nd}{F}\sin\theta \right]}^{1/2}-{\left[1+{\left(\frac{(j-N)d}{F}\right)}^{2}-2\frac{(j-N)d}{F}\sin\theta \right]}^{1/2}\right\}$$

In the formula, *c* is the wave speed in the medium; *d* is the distance between chips; *F* is the focal length; *N* is half of the number of excitation chips; *θ* is the deflection angle; and *j* is the wafer number.

## 3. Simulation and Experiment of Ultrasonic Phased Array for Seal Ring Groove

### 3.1. Detecting Objects

#### 3.1.1. Test Specimen

**Figure 6:**
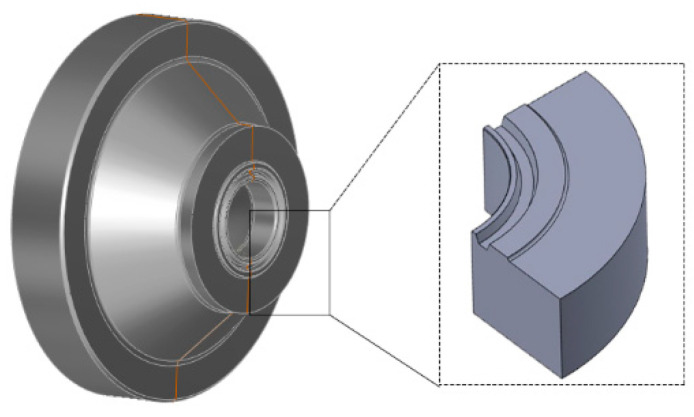
Three-dimensional schematic diagram of the test specimen.

A 25CrNiMo annular BOP FH35-35/70 gasket ring groove was selected for testing. Its three-dimensional structure is shown in Figure 6. The inside, surface, and near-surface of the BOP gasket ring groove may have pores generated during manufacturing. The residual slag in the BOP gasket ring groove weld after welding can reduce the plasticity and toughness of the weld. Weld cracking can cause BOP fracture. In this paper, ultrasonic phased array technology is used to detect defects, including pores, slag inclusions, and cracks, in the seal ring groove of the BOP.

#### 3.1.2. Artificial Defects

**Figure 7:**
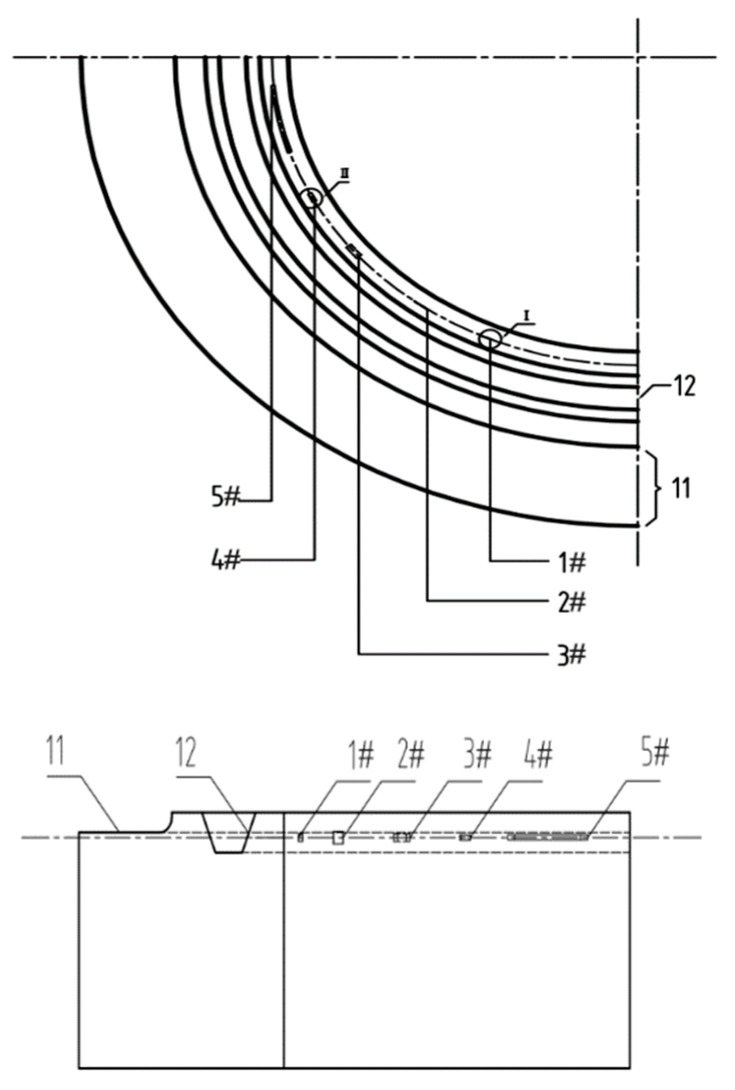
Schematic diagram of artificial defect processing on the test specimen.

In this study, two types of artificial defects, i.e., groove and transverse holes, were manufactured on the specimen. Artificial defects with different sizes were manufactured in different positions and directions on the specimen to simulate cracks, slag inclusions, and porosity defects in the gasket ring groove, as shown in Table 1. The distances from the center of the rightmost section of artificial defects 1# and 2# to the right vertical section of the test specimen body are 73 mm and 104 mm, and the dimensions are 5 × 3 × 0.2 mm and 8 × 5 × 0.2 mm, respectively, as shown in Figure 7. The distances from the center of the rightmost section of artificial defects 3#, 4#, and 5# to the vertical section of the rightmost section of the test specimen body are 138 mm, 160 mm, and 184 mm, and the dimensions are Φ 3 × 8 mm, Φ 2 × 5 mm, and Φ 2 × 40 mm, respectively. The vertical distance between all defects and the upper surface of the test specimen is 8 mm. The number 11 in Figure 7 is the plane part of the test specimen, and the number 12 is steel ring grooves.

### 3.2. Simulation of Ultrasonic Phased Array

First, the sound field of the probe in the natural state is theoretically analyzed, and the sound field coverage of the probe is verified by means of simulation to meet the detection requirement. The one-dimensional linear ultrasonic phased array transducer used in this process has a rectangular shape, and the sound pressure distribution on the central axis is different from that of an ordinary circular ultrasonic transducer. Even so, it still conforms to the rule that in the region where the distance is smaller than the length N of the near-field, the sound pressure exhibits multiple maxima and minima. When the distance is larger than N, the sound pressure decreases monotonically with increasing distance. The length of the ultrasonic phased array probe propagating in the workpiece should be subtracted from the length of the near-field of the radiated sound field and the equivalent length of the sound beam propagating in the wedge. To better analyze the relationship between the near-field area and the workpiece, the near-field depth (*N*_dep_) is commonly used to evaluate the sound field. The near-field depth can be expressed as:

(7) $$N_{\mathrm{dep}}=N\cos\beta$$

(8) $$N=0.35\frac{f}{c_{s}}{\left[A\frac{\cos\beta }{\cos\alpha }\right]}^{2}-L\frac{\tan\alpha }{\tan\beta }$$

where f is the frequency (MHz); *C_s_* is the shear wave velocity in the workpiece (m/s); *A* is the probe aperture (mm); *α* is the incidence angle of the sound beam; *β* is the refraction angle of the sound beam; and *L* is the propagation length of the sound beam in the wedge (mm). The parameters selected for this process are f = 5 MHz, *C_s_* = 3230 m/s, *A* = 16 mm, *α* = 36°, *β* = 55°, and *L* = 20 mm. By introducing the parameters into Equation (8), the length of the calculated near-field area is 59.54 mm, and the depth of the near-field area is 34.15 mm. The depth of the tested area of the test specimen is 30 mm; therefore, the selected probe can scan the entire tested area and be in the upper and lower coverage area of the maximum sound pressure to obtain ideal sensitivity and resolution.

**Figure 8:**
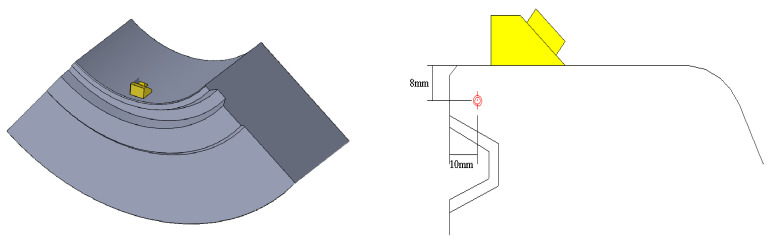
Probe arrangement and simulation.

Based on the finite difference model theory, this paper uses CIVA software for simulation, which can be applied to the modeling and analysis of various complex structure workpieces [30]. Among them, the two functions included in the ultrasonic inspection module are sound beam simulation and defect response. The former is convenient for test analysts to set appropriate inspection process parameters according to different situations, and the latter can simulate the response signal of defects [31,32]. Therefore, based on the finite element simulation platform CIVA, phased array ultrasonic testing is carried out to analyze the propagation characteristics of ultrasonication and then guide the actual detection, as shown in Figure 8.

### 3.3. Ultrasonic Phased Array Test

**Figure 9:**
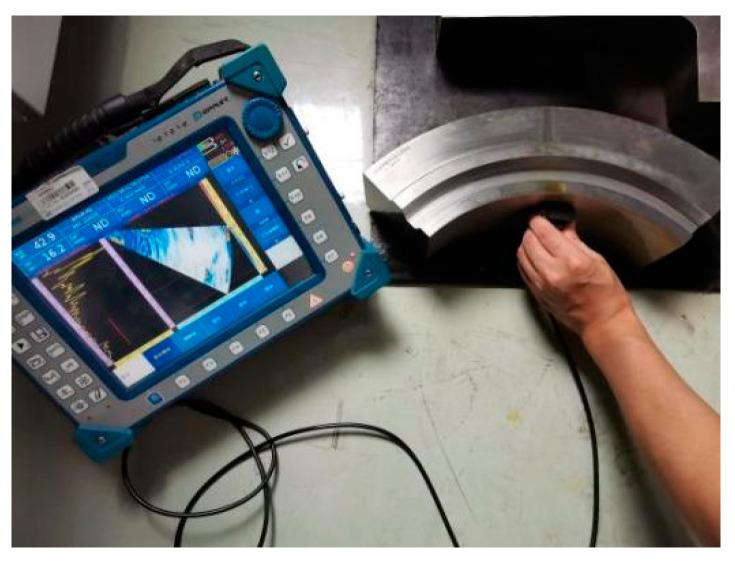
Ultrasonic phased array field test.

Ultrasonic phased array equipment mainly includes focusing rule generators, delay controllers, several independent transmitting and receiving channels, signal processors, and image processing and display modules. First, the delay rule is generated according to the instrument setting and scanning mode, and then the delay controller controls the time of each excitation probe chip according to the delay rule. The transmitting circuit generates the excitation voltage and loads it onto the corresponding phased array chip to generate an ultrasonic wave; then, the receiving circuit receives the ultrasonic signal received by each chip. The delay time of the signal processor is set up according to the law of synthesis of various chips of ultrasonic signals to obtain the delay rule corresponding to the A scanning signal. Thereafter, the ultrasonic phased array device will be an A sweep signal amplitude by color quantization, converting the A scanning signal into a color bar. Then, according to the scanning mode, the corresponding scan images are obtained, and, finally, the image is displayed on the screen. A PHSCAN phased array detector is used in the ultrasonic phased array test. The probe model is 5L32-0.5-10-D2; the wedge model is SD2-N55S-IHC; the wedge angle is 36°; and the sound beam delay is 14.94 μs. The process parameters were set according to the simulation results to achieve coverage of the probe to the detection range and ensure the ability of effective detection. The test sample in Figure 9 and its artificial defects were processed according to the design in Figure 7, and the sample material was 25CrNiMo. The cracks and inclusions are machined into the specimen and then surfacing processed. After machination, the sample is obtained.

## 4. Results Analysis and Discussion

### 4.1. Analysis of Simulation Results

**Figure 10:**
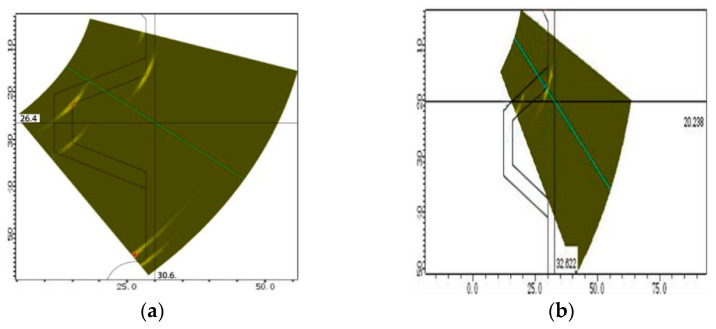
Echo analysis of the structure signal in the defect-free position of the test specimen. (**a**) Offset distance(>20 mm) (**b**) Offset distance(10–15 mm).

According to the thickness of the test block and the structure of the detection area, the detection frequency, crystal number, and probe aperture are determined. By changing the distance between the probe and the edge of the test block (offset distance) and the scanning angle, the offset distance and scanning angle that can cover the detection area are determined. When the adjusted offset distance is larger (>20 mm) and the scanning angle range is larger (38~75°), more structural echoes appear, which affects the judgment of defect echoes, as shown in Figure 10a. When the offset distance is 10–15 mm and the scanning angle is 40–70°, there are fewer structure echoes, and the defect echoes can be more accurately distinguished. We determined that the offset distance was 12 mm and the scanning angle was 40–70°, as shown in Figure 10b.

**Figure 11:**
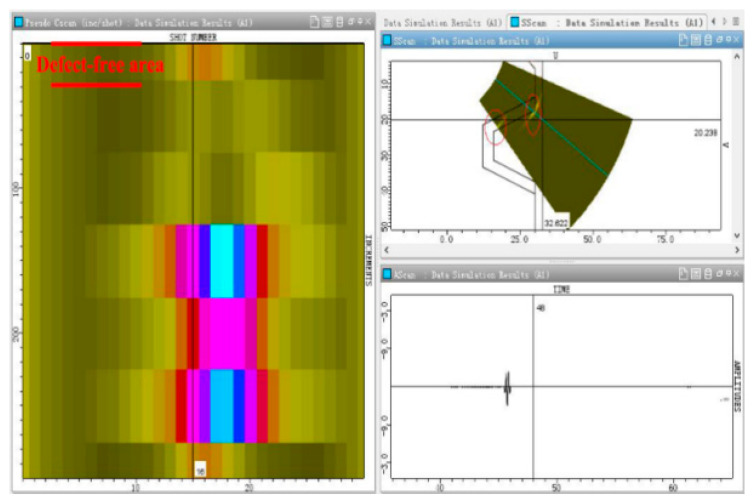
Simulation analysis of structural signal echo in the position without defect of the specimen.

Simulation software is used to simulate the ultrasonic phased array scanning specimen. The left image is the c-scanning result, the upper right image is the fan-scanning result, and the lower right image is the A-scanning result. As seen from the fan-scanning result, the specimen generates fixed geometric reflection signals at edges and corners at the top edges and corners of the cushion ring groove and inside the groove, respectively, when the position without defects is scanned. This is shown in the red circle marked in Figure 11.

**Figure 12:**
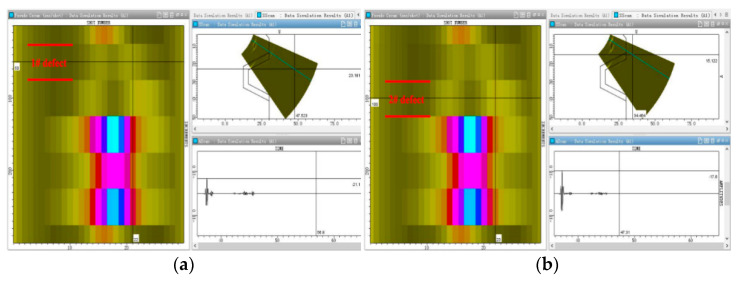
Simulation analysis of two kinds of crack defect signal echoes. (**a**) Defect 1#. (**b**) Defect 2#.

First, it was found that defect 1# with a simulated crack (5 × 3 × 0.2 mm) can be scanned by moving the probe in a circular direction along the inner wall of the main bore, as shown in Figure 12a. Two kinds of signal echoes can be clearly distinguished from the fan-scanning image; these are the structural signal echoes and the defect signal echoes. The horizontal and vertical distances of buried defects can be accurately calculated in the fan-scanning image to determine the position of defects in the workpiece. In addition, it can be seen in the A-scanning image that the defect signal echo appears before the structural signal echo, which can assist in the judgment of the defect. The image signal of defect 1# can be distinguished from the C-scanning image, but the signal is weak due to the small size of the crack defect. There is a significant increase in the defect signal in the C-scanning image when crack defect 2# (8 × 5 × 0.2 mm), whose size is larger than that of 1#, is detected, which is shown in Figure 12b. It can also be seen in the A-scanning image that the amplitude of the signal wave of crack defect 2# increases compared with that of crack defect 1#. At the same time, it can be seen in the fan-scanning image in Figure 12b that the structural signal echo and defect signal echo are also clearly distinguished.

**Figure 13:**
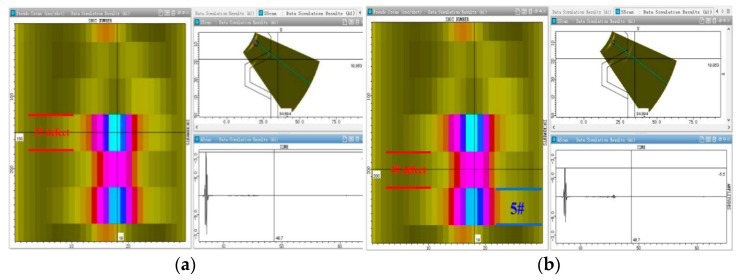
Simulation analysis of signal echoes of slag inclusions and porosity defects. (**a**) Defect 3#. (**b**) Defect 4#.

Figure 13a,b show ultrasonic phased array scanning images of defect 3# of the simulated slag inclusion and defect 4# of the simulated porosity, respectively. The echo of the defect signal in the fan-scanning image is also clearly distinguished from that of the structure signal. Moreover, as the vertical depth and horizontal distance of the four defects in the specimen are consistent, the feedback signal of the defect in the fan-scanning image is almost in the same position. As shown in Figure 13a,b, the signal of defect 3# simulated by slag inclusion and defect 4# simulated by porosity is significantly enhanced, which is due to the relatively large size of slag inclusion and porosity defects, resulting in a strong signal echo. However, the signal echo generated by defects 1# and 2# simulated by the crack is relatively weak due to the small size. In the C-scanning image, the signal echo below defect 4# is the echo of the calibration transverse hole.

According to the signal echo analysis, the signal amplitudes of defects 1#, 2#, 3#, and 4# are −21.1 dB, −17.6 dB, −1.8 dB, and −5.5 dB, respectively, and the reference amplitude of the transverse hole calibration is −2.1 dB, as shown in Table 2. For crack defects, the amplitude of defect signal increases from −21.1 dB to −17.6 dB, and the amplitude increases by 3.5 dB when the crack length increases from 5 mm to 8 mm; when the depth increases from 3 mm to 5 mm, the signal amplitude increases from −21.1 dB to −17.6 dB, and the amplitude increases by 3.5 dB. The results show that the signal amplitude increases with increasing crack length and depth [24]. For slag inclusion and porosity defects, the signal amplitude is obviously higher than that of crack defects.

### 4.2. Test Result Analysis

**Figure 14:**
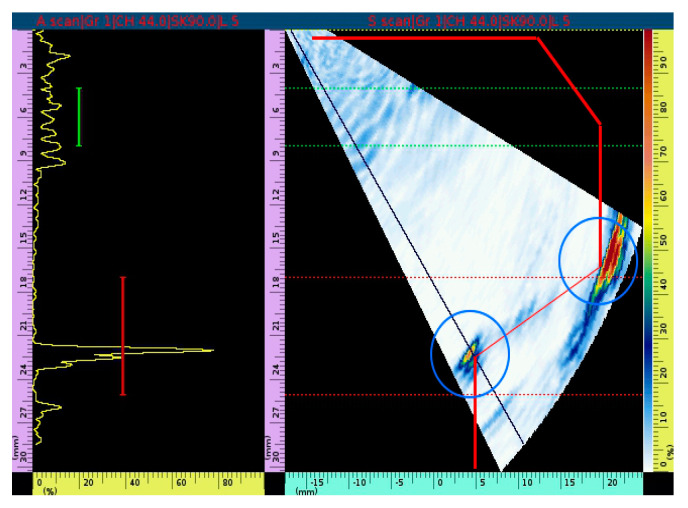
Echo analysis of the structure signal in the defect-free position of the test specimen.

According to the testing process parameters determined by simulation, ultrasonic phased array testing equipment was used for experimental analysis. The test results were compared to the simulation results to verify the rationality of the finite element model. The result of scanning the defect-free position of the specimen is shown in Figure 14. The left part of the figure is the A-scanning waveform image, and the right part is the fan-scanning image. Two structural signal echoes can be clearly seen (blue circles in the figure), and the vertical distances of the two edges are 15 mm and 22 mm. Through the field measurement of the detected workpiece, it is seen that the two echoes correspond to the edges at the top of the cushion ring groove and the edges in the groove in the simulation.

**Figure 15:**
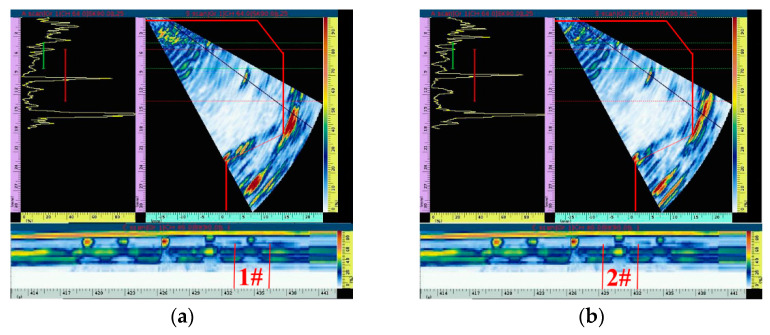
Signal echo analysis of two kinds of crack defects. (**a**) Defect 1#. (**b**) Defect 2#.

Circumferential scanning is used to detect defects. Figure 15a,b show the detection images of crack defects 1# and 2#, respectively. The A-scanning image is on the left, the fan-scanning image is on the right, and the C-scanning image is on the bottom. The signal echo of the internal crack defect and the signal echo of the workpiece structure can be clearly distinguished from the fan-scanning image. It can be seen from the simulation analysis that the signal echo of the crack defect is weak. In the actual detection process, it is necessary to adjust the input to identify the signal echo of the defect, which leads to many other signals, removing the signal echo of the defect and structure. These other signal echoes are the back wave of the detection contact surface and the secondary echo of the structure, which can also be clearly distinguished and excluded during the detection process. At the same time, the horizontal distance and vertical depth of the crack defect are 10.5 mm and 8.1 mm, respectively, which are basically consistent with the actual position of the defect inside the workpiece. In addition, it can also be seen in the C-scanning image that the signal echo of crack 2# is stronger than that of crack 1#, which is basically consistent with the simulation analysis result.

**Figure 16:**
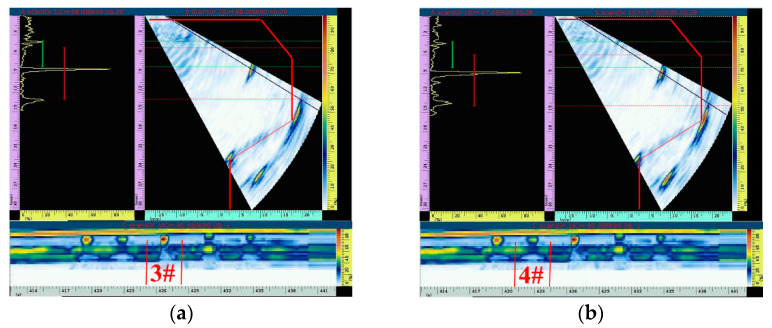
Signal echo analysis of slag inclusion and porosity defects. (**a**) Defect 3#. (**b**) Defect 4#.

**Figure 17:**
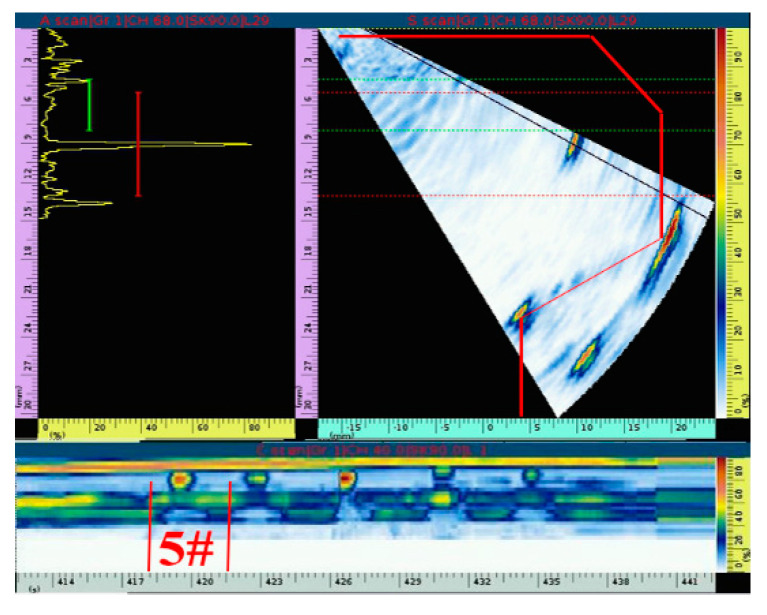
Signal echo analysis of the calibration hole.

The detection results of the simulated slag inclusion and porosity defects are shown in Figure 16a,b, respectively. As seen in the fan scan image, compared with the detection results of crack defects, the signal echoes of slag inclusion and porosity defects are stronger, resulting in fewer redundant signal echoes in the detection image, and it is easier to distinguish the structural signal echoes from the defect signal echoes. Similarly, the horizontal distance and vertical depth of slag inclusion and porosity defects are 10.2 mm and 7.8 mm, respectively, which are consistent with the actual position of defects inside the workpiece. The C-scanning image shows that the signal echo of the slag inclusion defect with the largest size is the strongest, followed by the porosity defect, which is also consistent with the simulation analysis result. In addition, the signal echo of the calibration hole is shown in Figure 17.

Using a comparative analysis between the field test and the simulation analysis, we found that the spot test based on the detection process parameters analyzed by the simulation can accurately determine the location of defects and identify the relative size of defects. This showed that the simulation analysis results and testing process parameters play a very good guiding role for the actual field test and testing.

**Figure 18:**
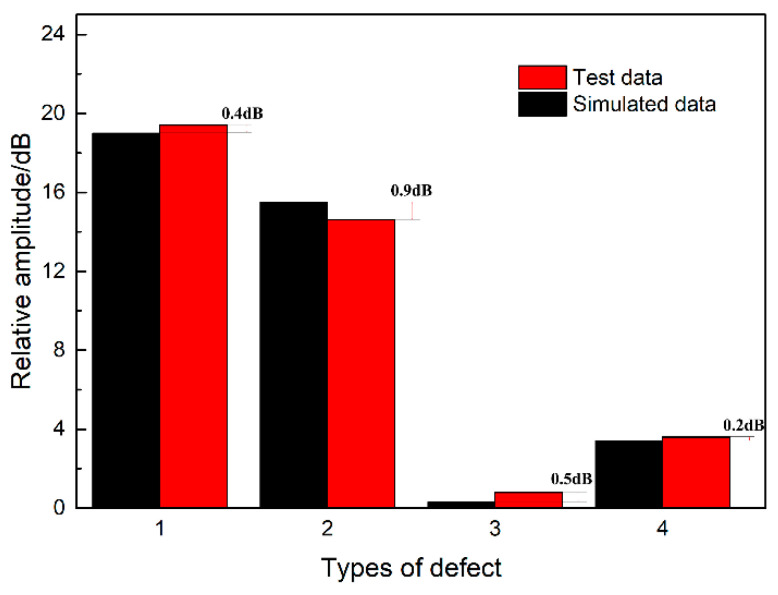
Comparison and analysis of test data and simulation data.

Furthermore, Table 3 shows that the relative amplitudes of the four defects in the test results compared with the calibration transverse hole are 19.4 dB, 14.6 dB, 0.3 dB, and 3.4 dB. The relative amplitude is basically consistent with the relative amplitude of the simulation results, and the difference of each item is within 1 dB, which also verifies the results of the simulation analysis, as shown in Figure 18.

## 5. Conclusions

In view of the complex structure of a seal ring groove and the influence of complex interference between the defect echo and the structural echo, the buried defects in the seal ring groove of a BOP are analyzed by combining ultrasonic phased array simulation analysis and actual test detection. The main research conclusions are as follows:

(1) The ultrasonic phased array detection technology can accurately identify the cracks, slag inclusions, and pores in the seal ring groove of a blowout preventer.

(2) The feedback signals of slag inclusions and pores generated during surfacing welding are obvious and can be easily detected. Buried cracks can also be detected, but the feedback signal is weak. With increasing crack length and depth size, the amplitude of the defect feedback signal increases. In view of the poor detection effect of microcracks, further measures are needed

(3) The relative amplitude of the test results is basically the same as that of the simulation results, and the difference is within 1 dB. Furthermore, the defect echo and structural echo can be clearly distinguished by the reasonable selection and arrangement of probes, such as a 5 MHz probe frequency, 36 array elements, and a 16 mm probe aperture, and by choosing the appropriate focal law. The results obtained in this study can provide a reference for actual field detection.

## Figures and Tables

**Table 1:** Artificial defect parameters.

Defect Number	Defect Type	Section Size/mm	Length/mm	Simulated Defect Type
1#	Groove	3	5	Simulated crack
2#	Groove	5	8	Simulated crack
3#	Horizontal hole	Ø 3	8	Simulated slag inclusion
4#	Horizontal hole	Ø 2	5	Simulated pore
5#	Horizontal hole	Ø 2	40	Calibration

**Table 2:** Analysis of simulation results of different types of defects.

Defect Number	Defect Size/mm	Amplitude/dB	Reference Amplitude/dB (2 × 40 Transverse Hole)	Relative Amplitude (Absolute Value)/dB
1#	5 × 3	−21.1	−2.1	19
2#	8 × 5	−17.6	−2.1	15.5
3#	8 × Ø 3	−1.8	−2.1	0.3
4#	5 × Ø 2	−5.5	−2.1	−3.4

**Table 3:** Analysis of the test results of different types of defects.

Defect Number	Defect Size/mm	Amplitude/dB	Reference Amplitude/dB (2 × 40 Transverse Hole)	Relative Amplitude (Absolute Value)/dB	Error/dB
1#	5 × 3	−46.9	−27.5	19.4	0.4
2#	8 × 5	−42.1	−27.5	14.6	0.9
3#	8 × Ø 3	−26.7	−27.5	0.8	0.5
4#	5 × Ø 2	−31.1	−27.5	3.6	0.2

## Data Availability

Not applicable.
